# Harmonization of adverse events monitoring following thoracic surgery: Pursuit of a common language and methodology

**DOI:** 10.1016/j.xjon.2021.03.021

**Published:** 2021-04-02

**Authors:** Gregory Sigler, Caitlin Anstee, Andrew J.E. Seely

**Affiliations:** a Division of General Surgery, Department of Surgery, University of Ottawa, Ottawa, Ontario, Canada; b Ottawa Hospital Research Institute, University of Ottawa, Ottawa, Ontario, Canada; c Division of Thoracic Surgery, Department of Surgery, University of Ottawa, Ottawa, Ontario, Canada

**Keywords:** thoracic surgery, adverse event monitoring, harmonization, Clavien–Dindo model, AE, adverse event, CATS, Canadian Association of Thoracic Surgeons, ECCG, Esophagectomy Complications Consensus Group, ESTS, European Society of Thoracic Surgeons, NSQIP, National Surgical Quality Improvement Program, STS, Society of Thoracic Surgeons, TM&M, Thoracic Morbidity and Mortality

## Abstract

**Objective:**

Thoracic surgery carries significant risk of postoperative adverse events (AEs). Multiple international recording systems are used to define and collect AEs following thoracic surgery procedures. We hypothesized that a simple-yet-ubiquitous approach to AE documentation could be developed to allow universal data entry into separate international databases.

**Methods:**

AE definitions of the Canadian Association of Thoracic Surgeons (CATS) system and 4 international databases were matched and compared. This consisted of reviewing the definition of each AE as described by their respective database and assessing compatibility with the CATS system. We developed a single set of 4 drop-down menus to enable clear classification and facilitated data entry, using 3 single-select mandatory lists and 1 multiselect optional list classifying type and severity of these events.

**Results:**

The CATS data elements were harmonized (ie, perfect or good) with 100% (European Society of Thoracic Surgeons), 89% (Society of Thoracic Surgeons), 74% (Esophagectomy Complications Consensus Group), and 73% (National Surgical Quality Improvement Program) of respective data elements. The addition of 17 AEs and 2 complication modifiers to the CATS system was implemented to achieve complete harmonization. Consequently, 100% of AE data elements currently included in all 4 international databases are perfectly or well-harmonized with the revised 4-choice drop down menu.

**Conclusions:**

We describe a framework for a ubiquitously applicable approach to AE monitoring following thoracic surgery harmonized with AE definitions of all major thoracic international associations. Use of this AE collection framework allows for comprehensive evaluation of both the incidence and severity of all AEs after thoracic surgery along with quality indicators.

## Graphical abstract

**Figure:**
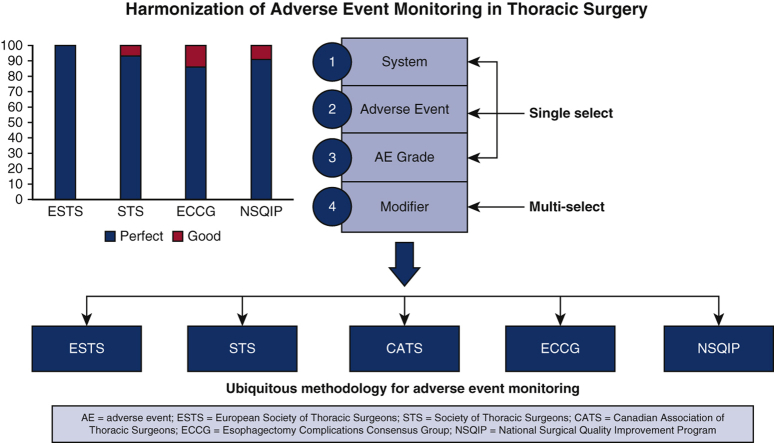

**Figure:**
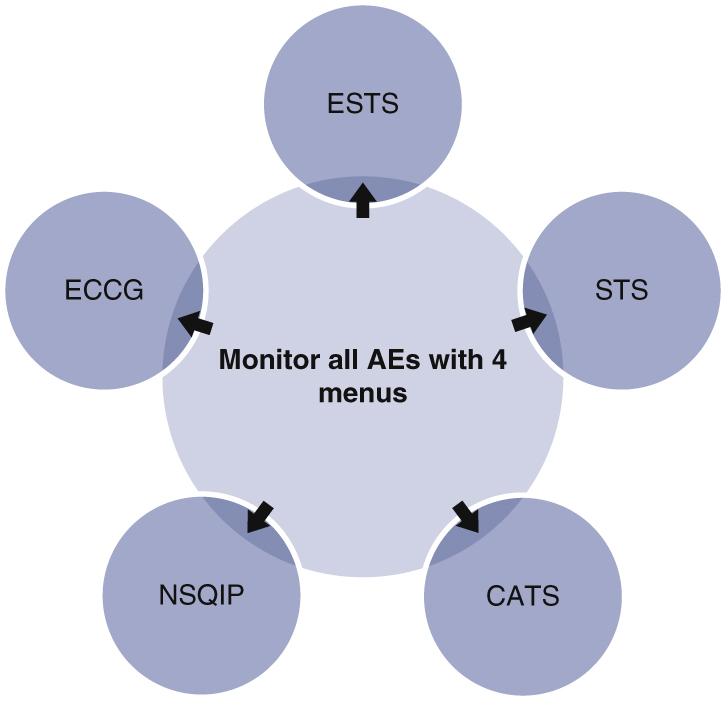
Four drop-down menus to classify postoperative adverse events.

**Central Message**

A ubiquitously applicable methodology using 4 drop-down menus to enable AE monitoring after thoracic surgery, harmonized with definitions of major thoracic international associations, is discussed.

**Perspective**

Multiple international recording systems are used to define and collect AEs following thoracic surgery procedures. A simple, ubiquitous approach to AE documentation was developed to allow universal data entry into separate international databases. This framework allows for comprehensive evaluation of both incidence and severity of all AEs after thoracic surgery, with potential for broad application.

See Commentary on page 257.

Thoracic surgery is the cornerstone of curative intent treatment for early-stage cancers of the chest, including lung and esophageal. However, it carries significant risk of postoperative adverse events (AEs), defined as any deviation from expected recovery from surgery, occurring in 30% to 60% of cases depending on the type of major thoracic surgery.^1^, ^2^, ^3^ AEs augment the risk of mortality,^3^^,^^4^ increase length of stay,^5^, ^6^, ^7^ lead to more readmissions,^8^^,^^9^ impair patient experience,^10^ and increases health care costs.^5^^,^^11^ In Canada, treatments for individual AEs range from $4000 to $12,000.^12^ While some AEs inevitably occur, between 37% and 51% of reported AEs are potentially preventable and cost the Canadian health care system $397 million/year.^7^^,^^12^ Comprehensive monitoring and documentation of AEs is therefore essential to inform clinical research and quality-improvement programs.

Multiple international recording systems are used to define and collect AEs following thoracic surgery procedures. These include the Society of Thoracic Surgeons (STS),^13^ the European Society of Thoracic Surgeons (ESTS),^14^ the Esophagectomy Complications Consensus Group (ECCG),^15^ the National Surgical Quality Improvement Program (NSQIP),^16^ and the Thoracic Morbidity and Mortality (TM&M) classification system^1^ adopted by the Canadian Association of Thoracic Surgeons (CATS). Increasingly, documentation of both severity and incidence of AEs has demonstrated enhanced capacity to track the impact of an AE in addition to its occurrence. The Clavien–Dindo classification system is a broadly applicable and validated tool used to track both incidence and severity of surgical complications based on the degree of therapeutic intervention required to treat the AE.^17^^,^^18^ Within thoracic surgery, the TM&M system is a standardized approach to identify both severity and incidence of thoracic-related postoperative AEs based on the Clavien–Dindo model.^1^ The TM&M model (https://www.ottawatmm.org) has demonstrated feasibility, reliability, and reproducibility as a tool for systematic monitoring, reporting, and evaluation of postoperative complications following thoracic surgery.^19^ It has been adopted by surgical groups internationally to gather prospective data regarding the burden of surgical complications and thus allows for continuous quality assessment.^20^^,^^21^ As it stands, there exists variation across systems in both which AEs are recorded and what criteria are used to describe these events. Although ESTS and STS have harmonized their AE definitions,^14^ they do not systematically measure AE incidence and severity. Substantial differences between NSQIP and TM&M data-collection systems exist, which call for improved harmonization, as well as inclusion of relevant thoracic-specific AE within the NSQIP system.^2^ This discordance limits the capacity to share information between databases. In addition, if a center wishes to participate in 2 international databases, it must collect both data separately. Improving capacity to simply record all AEs in a manner that it could be used for any international database would benefit the international research community interested in AE research and quality improvement.

We hypothesized that a simple-yet-ubiquitous approach to AE documentation could be developed that would allow universal data entry into the separate international databases. In other words, we sought to create a single AE-documentation system that could feed data to all systems, including STS, ESTS, TM&M, ECCG, and NSQIP. In this short paper, we introduce this system and formally evaluate whether this AE documentation and classification tool would enable ubiquitous and effective translation into other validated international AE classification systems. Our intention was to use this as a method to improve deficiencies in our own system, which we ultimately hope will allow improved collaboration among international research societies.

## Methods

**Figure 1:**
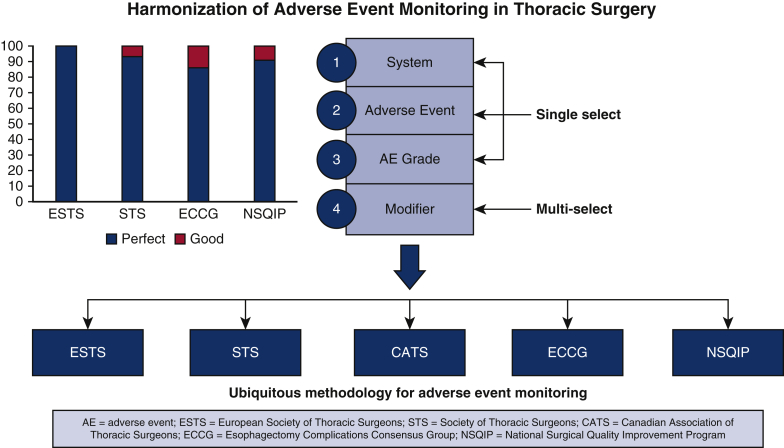
Our harmonized system uses a single set of 4 drop-down menus to classify postoperative adverse events (*AEs*), including system, adverse event, grade, and modifier. AEs may be fed into all international systems including the Canadian Association of Thoracic Surgeons (*CATS*), Society of Thoracic Surgeons (*STS*), European Society of Thoracic Surgeons (*ESTS*), Esophagectomy Complications Consensus Group (*ECCG*), and National Surgical Quality Improvement Program (*NSQIP*).

We aimed to create an AE classification system capable of harmonizing with all other database definitions. We developed a single set of 4 drop-down menus to enable clear classification and facilitated data entry (Figure 1). This system is labeled as the CATS system, as it has been adopted by that organization. The approach uses 3 single-select lists and 1 optional multiselect list to track the type and severity of these events. The single-select options include system (eg, pleural, pulmonary, gastrointestinal, neurologic, etc), type of complication (eg, pneumonia, air leak, etc), and Clavien–Dindo grade (eg, Grade I to V). Finally, the multiselect list includes modifiers (eg, resulted in prolonged length of stay, required emergency department visit, or hospital readmission, etc) that can be associated with each AE.

### AE Definitions

**Figure 2:**
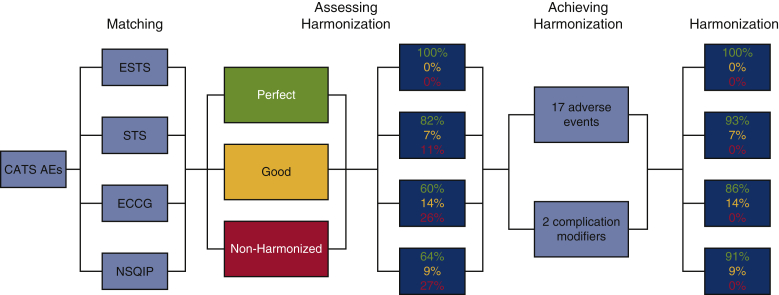
Flow diagram of the matching and harmonization process, read from left to right. The “Matching” process consisted of reviewing the definition of each adverse event (*AE*) as described by their respective database and assessing compatibility with those defined by the Canadian Association of Thoracic Surgeons' (*CATS*) system. “Assessing Harmonization” was accomplished by categorizing as Perfect (ie, exact wording), Good (ie, nearly exact wording), or Non-Harmonized the AE data elements of each classification system. The non-harmonized definitions were compiled and used to provide recommendations for prospective modifications to the CATS system (“Achieving Harmonization”), including the addition of 17 AEs and 2 complication modifiers. Following these modifications, 100% of AE data elements currently included in all international databases are perfectly or well harmonized with the revised four-drop down menu (“Harmonization”). *NSQIP*, National Surgical Quality Improvement Program; *ECCG*, Esophagectomy Complications Consensus Group; *STS*, Society of Thoracic Surgeons; *ESTS*, European Society of Thoracic Surgeons.

The AE definitions of the CATS system and those of ESTS, STS, ECCG, and NSQIP databases were matched and compared. The matching process consisted of reviewing the definition of each AE as described by their respective database and assessing compatibility with those defined by the CATS system (Figure 2). Any discrepancies between definitions and grades of AEs were explicitly noted. Tables were created to facilitate systems-based comparison (see Online Data Supplement 1). The degree to which AE data elements of the classification systems were harmonized with the CATS system were categorized as Perfect (ie, exact wording), Good (ie, nearly exact wording), or Non-Harmonized. The non-harmonized definitions were compiled and used to provide recommendations for prospective modifications to the CATS system (see Online Data Supplement 2).

### AE Collection

The methods of AE data collection not only require clear definitions that are harmonized across international groups but also necessitate prospective recording of AE on a daily basis, ensuring the treatment team is participating in the recording of AEs. AEs may be recorded using a set of 4 drop down menus (3 single-select and 1 multiselect). In addition to daily prospective entry, weekly review is recommended to highlight all AEs and their incidence and severity, ensuring multidisciplinary discussion regarding the labeling of AEs in both incidence and severity. This prospective daily entry and weekly discussion is recommended as optimal to capture all AEs after all surgeries at any institution.

## Results

**Figure 3:**
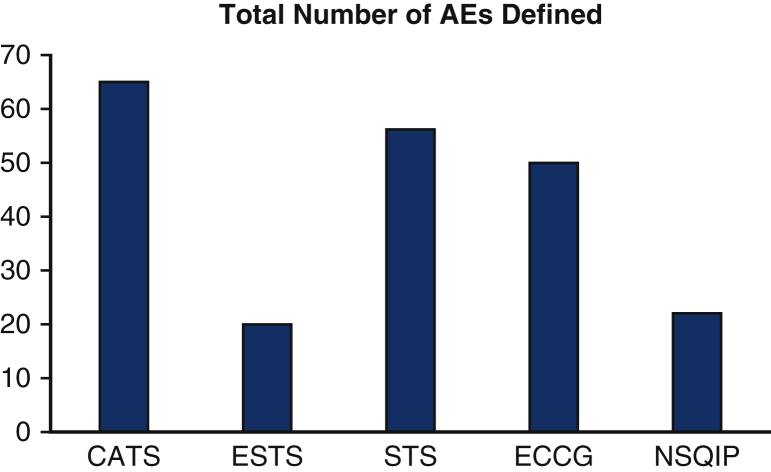
The number of postoperative adverse events (*AEs*) defined by each classification system: Canadian Association of Thoracic Surgeons (*CATS*), 65; European Society of Thoracic Surgeons (*ESTS*), 20; Society of Thoracic Surgeons (*STS*), 56; Esophagectomy Complications Consensus Group (*ECCG*), 50; National Surgical Quality Improvement Program (*NSQIP*), 22.

**Figure 4:**
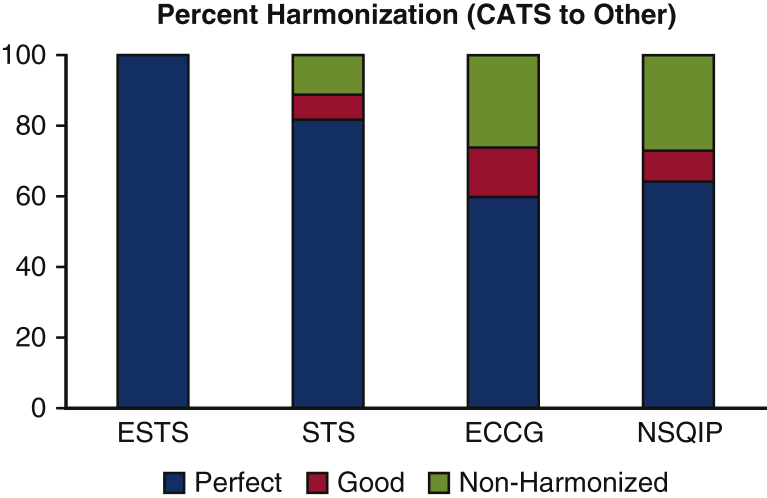
The initial matching process of the Canadian Association of Thoracic Surgeons (*CATS*) data elements with those of the European Society of Thoracic Surgeons (*ESTS*), Society of Thoracic Surgeons (*STS*), Esophagectomy Complications Consensus Group (*ECCG*), and National Surgical Quality Improvement Program (*NSQIP*). These were harmonized (ie, perfect or good) with 100%, 89%, 74%, and 73% for ESTS, STS, ECCG, and NSQIP, respectively. The 11%, 26%, and 27% of non-harmonized data elements for STS, ECCG, and NSQIP, respectively, represent adverse events not previously defined by the CATS system.

**Figure 5:**
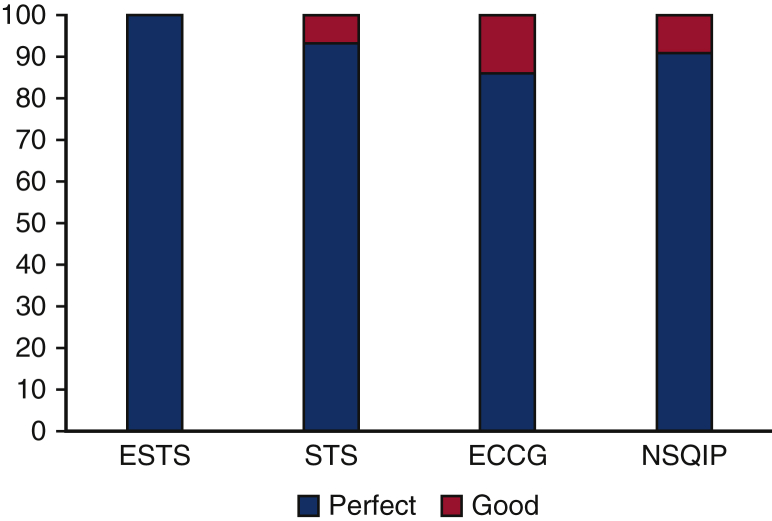
Following the addition of 17 postoperative adverse events and 2 complication modifiers to the Canadian Association of Thoracic Surgeons (CATS) system, it was perfectly harmonized with 100%, 93%, 86%, and 91% of the European Society of Thoracic Surgeons (*ESTS*), Society of Thoracic Surgeons (*STS*), Esophagectomy Complications Consensus Group (*ECCG*), and National Surgical Quality Improvement Program (*NSQIP*) data elements, respectively, with the remainder being well harmonized. The remaining terms that were not perfectly harmonized are those that were well-harmonized during the initial matching process, whose definitions could not be altered.

The total number of AEs defined in the CATS, ESTS, STS, ECCG, and NSQIP classification systems was 65, 20, 56, 50, and 22, respectively (Figure 3). The CATS data elements were harmonized (ie, perfect or good) with 100%, 89%, 74%, and 73% for ESTS, STS, ECCG, and NSQIP, respectively (Figure 4). Additional AEs from other classification systems that CATS had not defined were identified. To achieve complete harmonization, the following changes to the CATS system were proposed: the addition of 17 AEs and 2 complication modifiers (Table 1). These include AEs in multiple systems—cardiovascular (1), gastrointestinal (10), neurologic (1), other (3), and pulmonary (2)—and complication modifiers such as “Discharged with chest tube” and “discharged with home O2.” These additions resulted in the CATS TM&M system being perfectly harmonized with 100%, 93%, 86%, and 91% of ESTS, STS, ECCG, and NSQIP data elements, respectively, with the remainder being well harmonized (Figure 5). The remaining terms that were not perfectly harmonized are those that were well-harmonized during the initial matching process, whose definitions could not be altered. In summary, 100% of AE data elements currently included in all 4 international databases are perfectly or well harmonized with the revised 4-drop down menu.

**Table 1:** Proposed changes to the Canadian Association of Thoracic Surgeons (CATS) system to achieve complete harmonization with all other adverse event classification systems

System	Adverse event
Cardiovascular	Cardiac arrest requiring CPR
Gastrointestinal	C-Diff
Gastrointestinal	Feeding J-tube complication
Gastrointestinal	Liver dysfunction
Gastrointestinal	Pancreatitis
Gastrointestinal	Pyloromyotomy/pyloroplasty complication
Gastrointestinal	Small bowel obstruction
Gastrointestinal	Stricture
Gastrointestinal	Intra-abdominal bleeding
Gastrointestinal	Acute diaphragmatic hernia
Gastrointestinal	Intra-abdominal abscess
Other	Anemia or anemia requiring transfusion
Other	Bacteremia or bacteremic shock
Other	Central-line infection
Neurologic	Delirium tremens
Pulmonary	Acute aspiration
Pulmonary	Pulmonary abscess
Complication modifiers	Discharged with chest tube
	Discharged with home O2

A total of 17 adverse events and 2 complication modifiers were added. These include adverse events in multiple systems—cardiovascular (1), gastrointestinal (10), other (3), neurologic (1), and pulmonary (2)—and complication modifiers such as “discharged with chest tube” and “discharged with home O2.” *CPR*, Cardiopulmonary resuscitation; *C-Diff*, *Clostridium difficile*.

The recommendations for changes to the CATS classification tool were presented at the 2019 CATS National Database Meeting in April 2019. These recommendations were discussed, refined, and subsequently adopted by the CATS Database Governance Committee. Participants of the CATS national database began collecting data using these additional definitions as of July 2019.

## Discussion

Our comparison provides insight into the degree of similarities and differences between the AE monitoring systems studied. We developed a ubiquitous AE classification system with 4 drop-down menus and matched them with the other international databases. The ESTS tracks 20 AEs and matched 100% (20/20), which can be attributed to previous collaboration between the 2 societies.^10^ The STS definitions were considered 89% (50/56) perfect or good harmonization. This database identified 2 new AEs and 2 new complication modifiers (see Online Data Supplement 2). The ECCG definitions were far less harmonized at 74% (37/50), although this database identified 13 AEs now included in the CATS system (see Online Data Supplement 2). The NSQIP system did not uniquely identify any additional terms and was matched to 73% (16/22). There are 1, 1, and 2 instances in which 2 of 3 of these databases—STS, ECCG, and NSQIP, respectively—both identified a term not previously monitored by the TM&M system. There are 2 additional AEs that are monitored by all 3 of these databases, which have now been incorporated into the CATS system, which now include *Clostridium difficile* infection and bacteremia or bacteremic shock (sepsis, septic shock) as independent AEs. Thus, following the addition of these AEs, all AE recorded were well or perfectly harmonized with the STS, ESTS, ECCG, and NSQIP AE definitions. This capacity to create a ubiquitous data entry method in general demonstrates the broad applicability of the Clavien–Dindo classification schema adopted by TM&M and CATS. The utility of this ubiquitously applicable 4-drop down menu is that any vendor can see this information and thus feed data to ESTS, STS, CATS, NSQIP, and ECCG as they see fit. It can be adopted by any software tool and allow databases to be analyzed in a more synchronous manner.

Interestingly, our initial comparison of the databases showed that there were only 6 AEs (myocardial infarction, pulmonary embolism, cerebrovascular complication, pneumonia, acute renal failure, wound infection) and 1 complication modifier (requiring reintubation) that are universally defined by all 5 databases. A comparison of only CATS, ESTS, STS, and ECCG increases the total of universally defined AEs to 13, with the addition of atrial arrythmia, ventricular arrythmia, delirium, recurrent nerve palsy, chylothorax, atelectasis, and acute respiratory distress syndrome (Online Data Supplement 1). This further highlights the potential benefits of our model to the international research community interested in AE research and quality improvement. We have proposed a single AE documentation system that could feed data to all systems, including CATS, ESTS, STS, ECCG and NSQIP. The modified CATS system has become universally applicable to its four international counterparts. Our simple online tool allows the user to easily input data on 82 AEs and 10 complications modifiers in a straightforward and efficient manner. It holds an advantage over traditional methods of monitoring AEs in that it is an easily-accessible web-based platform that can be used by multiple stakeholders simultaneously and eliminates the need to fill out a lengthy case report form. While we do not currently have any formal evaluation of the time it would take to enter data, it would practically seem more efficient.

It is always a work in progress to review and maintain clearly defined AEs after thoracic surgery. One limitation in this work was the fact some AE definitions are not explicitly defined, which made determining harmonization challenging. As a small example, “deep vein thrombosis” is classified by ECCG as an AE; however, no specific criteria are provided to define this. In such cases, we considered the definition perfectly harmonized. This may have led to an overestimation in the degree of real-life harmonization if the CATS system with other databases.

The primary author (G.S.) performed a review of the data elements of each classification system. The elements whose definitions either matched exactly or whose meanings were clinically indistinguishable were considered be in “Perfect” harmonization. Any definition whose wording did not match exactly was reviewed with the supervising author (A.S.) to determine its degree of harmonization. The authors recognize that this process represents a limitation in our methodology, as our outcomes are based on 2 authors' opinions as opposed to a more extensive and rigorous evaluation of opinions of other surgeons and experts in the field. However, we feel that the definitions of each classification system as a whole are straightforward and intentionally highly objective.

The implementation of a ubiquitously applicable AE classification tool offers the ability to compare and share data with other international AE-monitoring systems. Broadening the volume of data available to assess surgical quality improvement has vast applications, including the study of more rare pathologies, procedures, and complications. The TM&M website reflects these changes (https://www.ottawatmm.org) and provides a tool to facilitate translation of corresponding data elements between classification systems. While our project draws a specific focus to AEs following thoracic surgery, we believe our work employs a simple strategy, which can be applied broadly to monitoring AEs following many types of surgical procedures. The goal of harmonization of data collection is not synonymous with joining together multiple databases. It is rather to ensure that AEs are simply collected in the same manner, capable of participating in any or all international data-collection systems.

## Conclusions

This paper provides a framework for discussion and advancement toward a harmonized approach for AE definition and recording following thoracic surgery (Video 1). We hope that our finding that a single set of 4 drop-down menus that can enable universal AE data collection is useful to surgeons and societies interested in broad international benchmarking. If AE data could be pooled across international databases, there will be greater to capacity to both understand the incidence and impact of AEs and enhance research and quality improvement programs to reduce or mitigate AEs.

### Conflict of Interest Statement

The authors reported no conflicts of interest.

The *Journal* policy requires editors and reviewers to disclose conflicts of interest and to decline handling or reviewing manuscripts for which they may have a conflict of interest. The editors and reviewers of this article have no conflicts of interest.

## Supplementary Data

Appendix 2

Video 1 Video recording of primary author (Gregory Sigler) describing the purpose, methodology, outcomes and implications of the project. Video available at: https://www.jtcvs.org/article/S2666-2736(21)00078-4/fulltext.
