# Supplementary material for: Harmonization of adverse events monitoring following thoracic surgery: Pursuit of a common language and methodology
Source: JTCVS Open. 2021 Apr 2;6:250–6. doi: 10.1016/j.xjon.2021.03.021 (PMC9390191; doi:10.1016/j.xjon.2021.03.021)
Supplement: Appendix 1 [file mmc1.pdf]

Table 1: AEs harmonized with CATS database

Classification System		CATS[1]	ESTS/STS[2]	STS 4.1[3]				ECCG[4]		NSQIP[5,6]		
Type of Complication	Grade	Complication	Complication	Complication				Complication		Complication		
Anastomotic		**Dehiscence/Conduit Failure**<br>Loss of integrity or viability of conduit due to ischemia or dehiscence										
	Grade I	This complication will not occur at this grade										
	Grade II	Medical therapy (e.g. antibiotics) added										
	Grade IIIa	Intervention required (e.g. opening of wound, percutaneous drainage) without GA						**Conduit necrosis/failure requiring surgery**<br>(Gastrointestinal)<br>*Type I:* Conduit necrosis focal identified endoscopically (Treatment—Additional monitoring or non-surgical therapy)				
	Grade IIIb	Surgical intervention performed under general anaesthesia		**Conduit Necrosis Requiring Surgery**<br>(Gastrointestinal)				*Type II* : Conduit necrosis focal identified endoscopically and not associated with free anastomotic or conduit leak<br>(Treatment—Surgical therapy not involving esophageal diversion)<br>*Type III* : Conduit necrosis extensive<br>(Treatment—Treated with conduit resection with diversion)				
	Grade IVa	Leads to single organ failure										
	Grade IVb	Leads to multiple organ failure										
	Grade V	Death										
		**Delayed Emptying**<br>Partial paralysis of the stomach resulting in food remaining in the stomach for a longer period of time than normal										
	Grade I	No intervention required										
	Grade II	Medical therapy (e.g. antibiotics) added		Indicate whether **delayed conduit emptying** required intervention such as pyloric dilation, botox injection, and/or maintenance of NG drainage for more than seven days<br>(Gastrointestinal)	**Gastric Outlet Obstruction**<br>Indicate whether the patient experienced a gastric outlet obstruction requiring intervention (e.g., IV for dehydration, endoscopy and dilation, reoperation, etc.) in the postoperative period			**Delayed gastric emptying** requiring intervention or delaying discharge or requiring maintenance of NG drainage >7 days post-op<br>(Gastrointestinal)				
	Grade IIIa	Intervention required (e.g. opening of wound, percutaneous drainage) without GA										
	Grade IIIb	Surgical intervention performed under general anaesthesia										
	Grade IVa	Leads to single organ failure										
	Grade IVb	Leads to multiple organ failure										
	Grade V	Death										
		**Leak**<br>Breakdown of a suture line in a surgical anastomosis with leakage of gastric or intestinal fluid		**Anastomotic Leak Following Esophageal Surgery**<br>Indicate if the patient had an anastomotic leak following esophageal surgery				**Esophagogastric Leak From Anastomosis, Staple Line or Localized Conduit Necrosis**<br>Full thickness GI defect involving esophagus, anastomosis, staple line, or conduit irrespective of presentation or method of identification<br>(Gastrointestinal)				
	Grade I	Transient or equivocal, no therapy added										
	Grade II	Medical therapy (e.g. antibiotics) added					**Anastomotic Leak Requiring Medical Treatment Only**<br>Indicate whether the patient experienced an esophageal anastomosis leak that required medical management only (i.e., interventional radiation (IR) drainage, NPO, antibiotics, etc.) If a leak occurs on Barium Swallow only and does not require surgical intervention/drainage, (i.e., treated with NPO and delay in oral intake), then code this element as "Yes"<br>(Gastrointestinal)	*Type I:* Local defect requiring no change in therapy or treated medically or with dietary modification				
	Grade IIIa	Intervention required (e.g. opening of wound, percutaneous drainage) without GA			**Anastomotic Leak - Stent Placement**<br>Indicate if stent placement was utilized for the anastomotic leak	**Anastomotic Leak - Additional Chest Tube Placement**<br>Indicate if additional chest tube placement was utilized for the anastomotic leak		*Type II* : Localized defect requiring interventional but not surgical therapy, for example, interventional radiology drain, stent or bedside opening, and packing of incision				
	Grade IIIb	Surgical intervention performed under general anaesthesia			**Anastomotic Leak - Surgical Drainage and Repair**<br>Indicate if surgical drainage and repair were utilized for the anastomotic leak			*Type III* : Localized defect requiring surgical therapy	**Reoperation for reasons other than bleeding, anastomotic leak or conduit necrosis** (Other)			
	Grade IVa	Leads to single organ failure										
	Grade IVb	Leads to multiple organ failure										
	Grade V	Death										
		**Other Anastomotic Event**										
	Grade I	Transient or equivocal, no therapy added										
	Grade II	Medical therapy added										
	Grade IIIa	Surgical, radiological, endoscopic intervention, or multi-therapy intervention (does not require general anaesthesia)		**Unanticipated Post-Operative Invasive Procedure**<br>Indicate if the patient had an unplanned invasive procedure after surgery. Examples includes return to the operating room for a redo surgical procedure, a percutaneous procedure performed at bedside or in the radiology suite, a tracheostomy, and wound opening at bedside<br>Exclusions: postoperative toilet bronchoscopy, central venous access, arterial line placement, foley catheter placement								
	Grade IIIb	Surgical intervention performed under general anaesthesia			**Other events requiring OR with general anesthesia**<br>Indicate whether the patient experienced any other surgical events in the post-operative period requiring a procedure with general anesthesia<br>(Miscellaneous)	**Unexpected Return to the OR**<br>Indicate whether the patient was unexpectedly returned to the OR during this hospital visit				**Unplanned Reoperation**<br>[REOPERATION1]<br>Patient had an unplanned return to the operating room for a surgical procedure related to either the index or concurrent procedure performed; This return must be within the 30 day postoperative period		
	Grade IVa	Leads to single organ failure										
	Grade IVb	Leads to multiple organ failure						**Multisystem Organ Failure**<br>(Other)				
	Grade V	Death										
Cardiovascular		**Atrial Arrhythmia**<br>New onset of atrial fibrillation/flutter (AF) requiring medical treatment or cardioversion; does not include recurrence of AF that was present preoperatively										
	Grade I	Converting after correction of electrolytes; no other intervention required										
	Grade II	Requiring medications (e.g. beta-blockers) for heart rate control	Atrial Arrhythmia	Atrial Arrhythmia Requiring Treatment				**Dysrhythmia atrial** requiring intervention<br>(Cardiac)				
	Grade IIIa	Symptomatic, requiring a cardio-version or a device (e.g., pacemaker)										
	Grade IIIb	Operative intervention required under general anaesthesia										
	Grade IVa	Associated with single organ failure (e.g. CHF, hypotension, syncope, shock)										
	Grade IVb	Concomitant multiorgan complications										
	Grade V	Death										
		**Cardiac Effusion or Pericarditis**<br>Presence of an abnormal amount of fluid in the pericardial space; inflammation of the pericardium, often with fluid accumulation										
	Grade I	No intervention required										
	Grade II	Medical therapy adequate										
	Grade IIIa	Requiring pericardio-centesis						**Pericarditis** requiring intervention<br>(Cardiac)				
	Grade IIIb	Require operative drainage										
	Grade IVa	Single-organ failure										
	Grade IVb	Concomitant multiorgan complications										
	Grade V	Death										
		**Cardiac Herniation Post Intra-Pericardial Resection**<br>Sudden cardiogenic shock caused by torsion of the heart										
	Grade I	This complication will not occur at this grade										
	Grade II	This complication will not occur at this grade										
	Grade IIIa	This complication will not occur at this grade										
	Grade IIIb	Requires surgical repair										
	Grade IVa	Single-organ dysfunction										
	Grade IVb	Concomitant multiorgan complications										
	Grade V	Death										
		**Congestive Heart Failure (CHF)**<br>Cardiac output is insufficient to meet the body's normal requirements for oxygen and nutrients, and pulmonary edema develops										
	Grade I	No intervention required										
	Grade II	Symptomatic and requiring additional medical therapy: diuretics, beta-blockers, nitrates, calcium channel blockers, ACE inhibitors, phosphor-diesterase inhibition, calcium blockers						**Congestive heart failure** requiring intervention<br>(Cardiac)				
	Grade IIIa	Symptomatic and requiring drugs and non-invasive ventilation (e.g. BIPAP) for < 24 hrs										
	Grade IIIb	Requires ventricular assist device, ventricular reduction surgery, or heart transplant indicated										
	Grade IVa	Symptomatic and requiring drugs and non-invasive ventilation (e.g. BIPAP) for > 24 hrs; or requiring intubation and ventilation										
	Grade IVb	Concomitant multiorgan complications										
	Grade V	Death										
		**Heart Block**<br>Partial or complete interruption of impulse transmission from the atria to the ventricles										
	Grade I	Asymptomatic, intervention not indicated										
	Grade II	Requiring medications (e.g. atropine)										
	Grade IIIa	Symptomatic, requiring a device (e.g. pacemaker)										
	Grade IIIb	Operative intervention required under general anaesthesia										
	Grade IVa	Associated with single organ failure (e.g. CHF, hypotension, syncope, shock)										
	Grade IVb	Concomitant multiorgan complications										
	Grade V	Death										
		**Hypertension (HTN)**<br>Generally symptomless medical condition in which the blood pressure is chronically elevated										
	Grade I	Defined as persistent BP mean > 80 mmHg). No intervention required										
	Grade II	Recurrent or persistent requiring one or more additional drug therapy										
	Grade IIIa	Requiring intensive care admission if malignant type HTN										
	Grade IIIb	Operative intervention required under general anaesthesia										
	Grade IVa	Single-organ failure										
	Grade IVb	Concomitant multiorgan complications										
	Grade V	Death										
		**Myocardial Ischemia**<br>Restriction in blood supply, due to factors in the blood vessels, leading to damage or dysfunction of tissue										
	Grade I	Elevation Troponin, asymptomatic										
	Grade II	Requiring angina medications (e.g. beta blockers, NITRO)										
	Grade IIIa	Requiring percutaneous coronary intervention (PCI)										
	Grade IIIb	Requires urgent cardiac surgery for aorto-coronary bypass grafting										
	Grade IVa	Associated with single organ failure (e.g. CHF, hypotension, syncope, shock)										
	Grade IVb	Concomitant multiorgan complications										
	Grade V	Death										
		**Myocardial Infarction**<br>Evidenced by one of the following criteria:<br>1. Transmural infarction diagnosed by the appearance of a new Q wave in two or more contiguous leads on ECG<br>2. Subendocardial infarction (non Q wave) evidenced by clinical, angiographic, electrocardiographic signs<br>3. Laboratory isoenzyme evidence of myocardial necrosis	Myocardial Infarction	Myocardial Infarction				**Myocardial Infarction**<br>(Cardiac)<br>i. Detection of rise and/or fall of cardiac biomarkers (preferably troponin) with at least one value above the 99th percentile of the upper reference limit together with evidence of myocardial ischaemia with at least one of the following:<br>a. symptoms of ischaemia (include various combinations of chest, upper extremity, jaw or epigastric discomfort with exertion or at rest; the discomfort usually lasts ≥20 min, often is diffuse, not localized, not positional, not affected by movement of the region and it may be accompanied by dyspnoea, diaphoresis, nausea or syncope);<br>b. ECG changes indicative of new ischaemia [new ST-T changes or new left bundle branch block (LBBB)–Minnesota codes: ST-depression 4.1; 4.2; ST-elevation 9.2; LBBB 7.1];<br>c. development of pathological Q waves in the ECG (Minnesota codes: 1.1.1 through 1.2.5 plus 1.2.7), including:<br>1. no unequivocal pathological Q waves in the first ECG or in event set of ECG(s) followed by a record with a pathological Q wave or<br>2. any Q wave in leads V2–V3 ≥ 0.02 s or QS complex in leads V2 and V3 or Q wave ≥0.03 s and ≥0.01 mV deep or QS complex in leads I, II, aVL, aVF or<br>3. V4–V6 in any two leads of a contiguous lead grouping (I, aVL, V6;V4–V6; II, III, aVF).<br>d. imaging evidence of new loss of viable myocardium or new regional wall motion abnormality		**Occurrences Myocardial Infarction**<br>[CDMI]<br>An acute myocardial infarction which occurred intraoperatively or within 30 days following surgery as manifested by one of the following:<br>Documentation of ECG changes indicative of acute MI (one or more of the following): ST elevation > 1 mm in two or more contiguous leads, New left bundle branch, New q-wave in two of more contiguous leads<br>New elevation in troponin greater than 3 times upper level of the reference range in the setting of suspected myocardial ischemia<br>Physician diagnosis of myocardial infarction		
	Grade I	Elevation Troponin, asymptomatic										
	Grade II	Requiring angina medications (e.g. beta blockers, NITRO)										
	Grade IIIa	Requiring percutaneous coronary intervention (PCI)										
	Grade IIIb	Requires urgent cardiac surgery for aorto-coronary bypass grafting										
	Grade IVa	Associated with single organ failure (e.g. CHF, hypotension, syncope, shock)										
	Grade IVb	Concomitant multiorgan complications										
	Grade V	Death										
		**Pulmonary Embolism**<br>Patient experienced a pulmonary embolus in the postoperative period as documented by a V/Q scan, angiogram, or spiral CT	Pulmonary Embolism	Pulmonary Embolism				**Pulmonary Embolism***<br>(Thromboembolic)		**Occurrences Pulmonary Embolism**<br>[PULEMBOL]<br>A pulmonary embolism must be noted within 30 days after the principal operative procedure AND the following criteria, A AND B below:<br>A. New diagnosis of a new blood clot in a pulmonary artery<br>AND<br>B. The patient has a V-Q scan interpreted as high probability of pulmonary embolism or a positive CT exam, TEE, pulmonary arteriogram, CT angiogram, or any other definitive imaging modality (including direct pathology examination such as autopsy).		
	Grade I	Non-occlusive, requires no therapy										
	Grade II	Requires anti-coagulation										
	Grade IIIa	Requires IVC filter										
	Grade IIIb	Requires surgical repair										
	Grade IVa	Single-organ dysfunction										
	Grade IVb	Concomitant multiorgan complications										
	Grade V	Death										
		**Venous Thrombosis**<br>Blood clot that forms within a vein						**Deep Vein Thrombosis***<br>(Thromboembolic)	**Peripheral Thrombophlebitis***<br>(Thromboembolic)	**Occurrences DVT/Thrombophlebitis**<br>[OTHDVT]<br>New diagnosis of blood clot or thrombus within the venous system (superficial or deep) which may be coupled with inflammation and requires treatment. Must be noted within 30 days after the principal operative procedure AND one of the following A or B below:<br>A. New Diagnosis of a [new] venous thrombosis (superficial or deep), confirmed by a duplex, venogram, CT scan, or any other definitive imaging modality (including direct pathology examination such as autopsy) AND the patient must be treated with anticoagulation therapy and/or placement of a vena cava filter or clipping of the vena cava, or the record indicates that treatment was warranted but there was no additional appropriate treatment option available.<br>B. As per (A) above, but the patient or decisionmaker has refused treatment. There must be documentation in the medical record of the [patient's] refusal of treatment.		
	Grade I	Non-occlusive, requires no therapy										
	Grade II	Requires anti-coagulation		**Deep Vein Thrombosis (DVT) Requiring Treatment**<br>Indicate whether the patient has experienced a DVT confirmed by doppler study, contrast study, or other study that required treatment<br>(Cardiovascular)								
	Grade IIIa	Requires IVC filter										
	Grade IIIb	Requires surgical repair										
	Grade IVa	Single-organ dysfunction										
	Grade IVb	Concomitant multiorgan complications										
	Grade V	Death										
		**Ventricular Arrhythmia**<br>Sustained ventricular tachycardia or ventricular fibrillation that has been clinically documented and treated by ablation therapy, implantable cardioverter defibrillator, permanent pacemaker, pharmacologic treatment, or cardioversion										
	Grade I	Converting after correction of electrolytes; no other intervention required										
	Grade II	Requiring medications (e.g. beta-blockers) for heart rate control	Ventricular Arrhythmia	Ventricular Arrhythmia Requiring Treatment				**Dysrhythmia ventricular** requiring intervention (Cardiac)				
	Grade IIIa	Symptomatic, requiring a cardio-version or a device (e.g., pacemaker)										
	Grade IIIb	Operative intervention required under general anaesthesia										
	Grade IVa	Associated with single organ failure (e.g. CHF, hypotension, syncope, shock)										
	Grade IVb	Concomitant multiorgan complications										
	Grade V	Death										
		**Other Cardiovascular Event**		**Other Cardiovascular Event**<br>Indicate whether any other CV event occurred including distal arterial embolism in the postoperative period								
	Grade I	Transient or equivocal, no therapy added										
	Grade II	Medical therapy added										
	Grade IIIa	Surgical, radiological, endoscopic intervention, or multi-therapy intervention (does not require general anaesthesia)			**Unanticipated Post-Operative Invasive Procedure**<br>Indicate if the patient had an unplanned invasive procedure after surgery. Examples includes return to the operating room for a redo surgical procedure, a percutaneous procedure performed at bedside or in the radiology suite, a tracheostomy, and wound opening at bedside<br>Exclusions: postoperative toilet bronchoscopy, central venous access, arterial line placement, foley catheter placement							
	Grade IIIb	Surgical intervention performed under general anaesthesia				**Other events requiring OR with general anesthesia**<br>Indicate whether the patient experienced any other surgical events in the post-operative period requiring a procedure with general anesthesia<br>(Miscellaneous)	**Unexpected Return to the OR**<br>Indicate whether the patient was unexpectedly returned to the OR during this hospital visit			**Unplanned Reoperation**<br>[REOPERATION1]<br>Patient had an unplanned return to the operating room for a surgical procedure related to either the index or concurrent procedure performed; This return must be within the 30 day postoperative period		
	Grade IVa	Leads to single organ failure										
	Grade IVb	Leads to multiple organ failure						**Multisystem Organ Failure**<br>(Other)				
	Grade V	Death										
Gastrointestinal		**Constipation**<br>Difficult or infrequent passage of stool, hardness of stool, or a feeling of incomplete evacuation										
	Grade I	No intervention required										
	Grade II	Requiring nasogastric intubation, use of additional laxatives, dietary modification, or enema										
	Grade IIIa	Obstipation with manual evacuation indicated										
	Grade IIIb	Surgical intervention performed under general anaesthesia										
	Grade IVa	Life-threatening consequences (e.g., obstruction, toxic megacolon)										
	Grade IVb	Concomitant multiorgan complications										
	Grade V	Death										
		**GI Bleeding**<br>Bleeding that starts in the gastrointestinal tract										
	Grade I	This complication will not occur at this grade										
	Grade II	Transfusion, PPI infusion, or other medical therapy						**GI bleeding** requiring intervention or transfusion<br>(Gastrointestinal)				
	Grade IIIa	Requires endoscopy										
	Grade IIIb	Surgical intervention performed under general anaesthesia										
	Grade IVa	Life-threatening; debilitating; organ failure present										
	Grade IVb	Concomitant multiorgan complications										
	Grade V	Death										
		**Ileus**<br>Disruption of the normal propulsive gastrointestinal motor activity										
	Grade I	No intervention required										
	Grade II	NPO. Requiring nasogastric intubation. IV fluids indicated > 24 hrs		**Ileus**<br>Indicate whether the patient experienced an ileus lasting > 3 days as defined by limited GI motility requiring treatment (e.g., nasogastric tube insertion for decompression, etc.) in the postoperative period<br>(Gastrointestinal)				**Ileus** requiring intervention<br>(Gastrointestinal)				
	Grade IIIa	Tube feedings, or TPN indicated > 24 hrs										
	Grade IIIb	Surgical intervention performed under general anaesthesia										
	Grade IVa	Life-threatening; debilitating; organ failure present										
	Grade IVb	Concomitant multiorgan complications										
	Grade V	Death										
		**Infectious Diarrhea**<br>Infection of the digestive system by a bacterium, virus, or parasite that results in frequent bowel motions producing excessive amounts of liquidy feces										
	Grade I	This complication will not occur at this grade										
	Grade II	Requiring antibiotics										
	Grade IIIa	Endoscopic, radiological or bedside interventions performed										
	Grade IIIb	Surgical intervention performed under general anaesthesia										
	Grade IVa	Life-threatening; debilitating; organ failure present										
	Grade IVb	Concomitant multiorgan complications										
	Grade V	Death										
		**Nausea**<br>Unpleasant feeling of needing to vomit										
	Grade I	No intervention required										
	Grade II	Requiring nasogastric intubation, and prokinetics (e.g. Maxeran, Gravol)										
	Grade IIIa	Tube feedings, nasogastric intubation, or TPN indicated ≥ 24 hrs										
	Grade IIIb	Surgical intervention performed under general anaesthesia										
	Grade IVa	Life-threatening; debilitating; organ failure present										
	Grade IVb	Concomitant multiorgan complications										
	Grade V	Death										
		**Non-Infectious Diarrhea**<br>Increase in the volume, wateriness, or frequency of bowel movements										
	Grade I	This complication will not occur at this grade										
	Grade II	Requiring anti-diarrhoeal treatment										
	Grade IIIa	Endoscopic, radiological or bedside interventions performed										
	Grade IIIb	Surgical intervention performed under general anaesthesia										
	Grade IVa	Life-threatening; debilitating; organ failure present										
	Grade IVb	Concomitant multiorgan complications										
	Grade V	Death										
		**Vomiting**<br>Expulsion of the contents of one's stomach through the mouth										
	Grade I	No intervention required										
	Grade II	NPO. Requiring nasogastric intubation, and prokinetics (e.g. Maxeran, Gravol)										
	Grade IIIa	Tube feedings, nasogastric intubation, rectal intubation, or TPN indicated ≥ 24 hrs										
	Grade IIIb	Surgical intervention performed under general anaesthesia										
	Grade IVa	Life-threatening; debilitating; organ failure present										
	Grade IVb	Concomitant multiorgan complications										
	Grade V	Death										
		**Other Gastrointestinal Event**		**Other GI Event**<br>Indicate if the patient experienced any other GI events in the postoperative period								
	Grade I	Transient or equivocal, no therapy added										
	Grade II	Medical therapy added										
	Grade IIIa	Surgical, radiological, endoscopic intervention, or multi-therapy intervention (does not require general anaesthesia)			**Unanticipated Post-Operative Invasive Procedure**<br>Indicate if the patient had an unplanned invasive procedure after surgery. Examples includes return to the operating room for a redo surgical procedure, a percutaneous procedure performed at bedside or in the radiology suite, a tracheostomy, and wound opening at bedside<br>Exclusions: postoperative toilet bronchoscopy, central venous access, arterial line placement, foley catheter placement							
	Grade IIIb	Surgical intervention performed under general anaesthesia				**Other events requiring OR with general anesthesia**<br>Indicate whether the patient experienced any other surgical events in the post-operative period requiring a procedure with general anesthesia<br>(Miscellaneous)	**Unexpected Return to the OR**<br>Indicate whether the patient was unexpectedly returned to the OR during this hospital visit			**Unplanned Reoperation**<br>[REOPERATION1]<br>Patient had an unplanned return to the operating room for a surgical procedure related to either the index or concurrent procedure performed; This return must be within the 30 day postoperative period		
	Grade IVa	Leads to single organ failure										
	Grade IVb	Leads to multiple organ failure						**Multisystem Organ Failure**<br>(Other)				
	Grade V	Death										
Neu[illegible]		**Cerebrovascular Complications**<br>Occurrence of one of the following central neurologic postoperative events not present preoperatively:<br>1. A central neurologic deficit persisting postoperatively for more than 72 hours<br>2. A transient neurologic deficit (transient ischemic attack or reversible ischemic neurologic deficit) with recovery within 72 hours<br>3. A new postoperative coma persisting at least 24 hours and caused by anoxic/ischemic and/or metabolic encephalopathy, thromboembolic event, or cerebral bleed	Cerebrovascular Complications	**New Central Neurological Event**<br>(Neurology)				**Stroke (CVA)***<br>(Thromboembolic)		**CVA/Stroke** with neurological deficit<br>[CNSCVA]<br>Patient develops an embolic, thrombotic, or hemorrhagic vascular accident or stroke with motor, sensory, or cognitive dysfunction (e.g., hemiplegia, hemiparesis, aphasia, sensory deficit, impaired memory) that persists for 24 or more hours. If a specific time frame for the dysfunction is not documented in the medical record, but there is a diagnosis of a stroke, assign the occurrence, unless documentation specifically states that the motor, sensory, or cognitive dysfunction resolved.		
	Grade I	No intervention required										
	Grade II	Requiring medical intervention (e.g. anticoagulants, ASA)										
	Grade IIIa	Radiological or bedside interventions performed										
	Grade IIIb	Surgical intervention performed										
	Grade IVa	Life-threatening, requiring intensive-care										
	Grade IVb	Concomitant multiorgan complications										
	Grade V	Death										
		**Confusion/Delirium**<br>Patient experienced a new onset of symptoms like illusions, confusion, cerebral excitement in the postoperative period	Delirium	**Delirium**<br>Indicate whether the patient experienced delirium in the postoperative period marked by illusions,<br>confusion, cerebral excitement, and having a comparatively short course<br>(Neurology)				**Acute Delirium**<br>(Neurologic/Psychiatric)<br>DSM-IV Criteria for Delirium<br>1. Disturbance of consciousness (i.e., reduced clarity of awareness of the environment) with reduced ability to focus, sustain or shift attention (yes/no)<br>2. A change in cognition or the development of a perceptual disturbance that is not better accounted for by a preexisting, established or evolving dementia (yes/no)<br>3. The disturbance develops over a short period of time (usually hours to days) and tends to fluctuate during the course of the day (yes/no)<br>4. There is evidence from the history, physical examination or laboratory findings that the disturbance is caused by the direct physiological consequences of a general medical condition (yes/no)				
	Grade I	Transient. New or worsened; no intervention required										
	Grade II	Requiring medical intervention										
	Grade IIIa	Prolonged hospitalization indicated; danger to self or others										
	Grade IIIb	This complication will not occur at this grade										
	Grade IVa	This complication will not occur at this grade										
	Grade IVb	This complication will not occur at this grade										
	Grade V	Death										

rological		**Recurrent Nerve Palsy**<br>Patient experienced in the postoperative period a recurrent laryngeal nerve paresis or paralysis that was not identified during the preoperative evaluation	Recurrent Nerve Palsy	**Recurrent Laryngeal Nerve Paresis**<br>Indicate whether the patient experienced in the postoperative period, paresis or paralysis of the recurrent laryngeal nerve<br>(Neurology)				**Recurrent Laryngeal Nerve Injury**<br>Vocal cord dysfunction post-resection. Confirmation and assessment should be by direct examination<br>(Neurologic/Psychiatric)<br>Severity Level<br>(A) Unilateral (B) Bilateral<br>For example, a unilateral vocal cord injury requiring elective medialization procedure (Final Type IIA)		
	Grade I	No intervention required						*Type I* : Transient injury requiring no therapy; Dietary modification allowed		
	Grade II	Medical therapy added								
	Grade IIIa	Endoscopic or radiological intervention performed								
	Grade IIIb	Surgical intervention performed under general anaesthesia						*Type II* : Injury requiring elective surgical procedure, for example, thyroplasty or medialization procedure<br>*Type III* : Injury requiring acute surgical intervention (due to aspiration or respiratory issues), for example, thyroplasty or medialization procedure		
	Grade IVa	Leads to single organ failure								
	Grade IVb	Leads to multiple organ failure								
	Grade V	Death								
		**Seizure**<br>Abnormal electrical discharge that occurs within the brain's cortical gray matter and interrupts normal brain function; maybe focal or generalized								
	Grade I	This complication will not occur at this grade								
	Grade II	Requiring medical intervention (e.g. anti-convulsants)								
	Grade IIIa	Poorly controlled seizure disorder, with breakthrough generalized seizures despite medical intervention								
	Grade IIIb	This complication will not occur at this grade								
	Grade IVa	Status epilepticus, intractable epilepsy, requiring mechanical ventilation								
	Grade IVb	This complication will not occur at this grade								
	Grade V	Death								
		**Other Neurological Event**		**Other Neurological Event**<br>Indicate whether the patient experienced any other neurologic event in the postoperative period				**Other Neurologic Injury**<br>(Neurologic/Psychiatric)		
	Grade I	Transient or equivocal, no therapy added								
	Grade II	Medical therapy added								
	Grade IIIa	Surgical, radiological, endoscopic intervention, or multi-therapy intervention (does not require general anaesthesia)			**Unanticipated Post-Operative Invasive Procedure**<br>Indicate if the patient had an unplanned invasive procedure after surgery. Examples includes return to the operating room for a redo surgical procedure, a percutaneous procedure performed at bedside or in the radiology suite, a tracheostomy, and wound opening at bedside<br>Exclusions: postoperative toilet bronchoscopy, central venous access, arterial line placement, foley catheter placement	**Other events requiring OR with general anesthesia**<br>Indicate whether the patient experienced any other surgical events in the post-operative period requiring a procedure with general anesthesia<br>(Miscellaneous)	**Unexpected Return to the OR**<br>Indicate whether the patient was unexpectedly returned to the OR during this hospital visit			**Unplanned Reoperation**<br>[REOPERATION1]<br>Patient had an unplanned return to the operating room for a surgical procedure related to either the index or concurrent procedure performed; This return must be within the 30 day postoperative period
	Grade IIIb	Surgical intervention performed under general anaesthesia								
	Grade IVa	Leads to single organ failure								
	Grade IVb	Leads to multiple organ failure							**Multisystem Organ Failure**<br>(Other)	
	Grade V	Death								
Pleural		**Bronchopleural Fistula**<br>Abnormal communication between a lobar or segmental bronchus and the pleural cavity (documented on bronchoscopy or at OR)	**Bronchopleural Fistula**<br>Patient experienced a complete or partial dehiscence of the bronchial stump documented in the postoperative period (such as bronchoscopy or other operative intervention)	**Bronchopleural Fistula**<br>Indicate if the patient experienced a documented bronchopleural fistula in the postoperative period. Bronchopleural fistula is defined as a major bronchial air leak requiring intervention such as a chest tube, operation, or other procedure<br>(Pulmonary)						
	Grade I	This complication will not occur at this grade								
	Grade II	This complication will not occur at this grade								
	Grade IIIa	Symptomatic; requiring: tube thoracostomy, endoscopic (e.g., stent), closure								
	Grade IIIb	Operative intervention with thoraco-plasty, chronic open drainage or multiple thoraco-tomies indicated								
	Grade IVa	Life-threatening; debilitating; organ failure present								
	Grade IVb	Concomitant multiorgan complications								
	Grade V	Death								
		**Chylothorax**<br>Patient experienced a chylothorax in the postoperative period that required persistent or new drainage and medical intervention (eg, NPO, TPN) or reoperation. Chylothorax is defined by the clinical appearance of the pleural fluid or the presence of pleural fluid triglyceride levels >110 mg/dL with a cholesterol level <200 mg/dL	Chylothorax	**Chylothorax**<br>Indicate if the patient had a chylothorax				**Chyle Leak**<br>(Other)<br>Severity Level<br>(A) <1 liter output/day (B) >1 liter output/day<br>For example, chyle leak initially producing 1200 ml/day and successfully treated by stopping enteric feeds and initiating TPN (Final Type IIB)		
	Grade I	This complication will not occur at this grade								
	Grade II	Requiring medical therapy (e.g. TPN, drainage for 7 days)			**Chylothorax Requiring Medical Intervention**<br>Indicate whether the patient experienced a chylothorax in the postoperative period that required medical intervention (i.e., NPO, TPN, etc.)			*Type I* : Treatment—Enteric dietary modifications<br>*Type II* : Treatment—Total parenteral nutrition		
	Grade IIIa	Require TPN, or endoscopic, radiological or bedside pleural interventions performed			**Chylothorax - Thoracic Duct Embolization Attempted**<br>Indicate if thoracic duct embolization was attempted for chylothorax				*Type III* : Treatment—interventional or surgical therapy	
	Grade IIIb	Surgical intervention performed under general anaesthesia			**Chylothorax Requiring Surgical Ligation of Thoracic Duct**<br>Indicate if the chylothorax required surgical ligation of the thoracic duct					
	Grade IVa	Life-threatening; debilitating; organ failure present								
	Grade IVb	Concomitant multiorgan complications								
	Grade V	Death								
		**Effusion**<br>Collection of fluid that accumulates in the pleural cavity								
	Grade I	Asymptomatic, no intervention indicated								
	Grade II	Requiring medical therapy (e.g. diuretics) for CHF; drainage is required if postoperative effusion is large								
	Grade IIIa	Endoscopic, radiological or bedside pleural interventions performed		**Post-op Pleural Effusion Requiring Drainage**<br>Indicate whether a postoperative pleural effusion required drainage via thoracentesis or chest tube insertion<br>(Pulmonary)				**Pleural effusion** requiring additional drainage procedure<br>(Pulmonary)		
	Grade IIIb	Surgical intervention performed under general anaesthesia								
	Grade IVa	Life-threatening; debilitating; organ failure present								
	Grade IVb	Concomitant multiorgan complications								
	Grade V	Death								
		**Empyema**<br>Patient experienced an empyema requiring treatment in the postoperative period; diagnosis of empyema should be confirmed by thoracentesis; frank pus or merely cloudy fluid may be aspirated from the pleural space; the pleural fluid typically has leukocytosis, low pH (<7.20), low glucose (<60 mg/dL), high lactate dehydrogenase, and elevated protein and may contain infectious organisms	Empyema							
	Grade I	This complication will not occur at this grade								
	Grade II	Requiring medical therapy (e.g. antibiotics)								
	Grade IIIa	Endoscopic, radiological or bedside pleural interventions performed		**Empyema Requiring Treatment**<br>Indicate whether the patient experienced an empyema requiring treatment in the postoperative period (i.e., chest tube drainage by interventional radiology, etc.)<br>(Infection)						
	Grade IIIb	Surgical intervention performed under general anaesthesia								
	Grade IVa	Life-threatening; debilitating; organ failure present								
	Grade IVb	Concomitant multiorgan complications								
	Grade V	Death								
		**Hemothorax**<br>Blood in the pleural cavity								
	Grade I	This complication will not occur at this grade								
	Grade II	Requiring medical therapy. Requiring drainage at >300 cc/hr for 3 hrs								
	Grade IIIa	Endoscopic, radiological or bedside pleural interventions performed						Reoperation for **thoracic bleeding**<br>(Other)		
	Grade IIIb	Surgical intervention performed under general anaesthesia								
	Grade IVa	Life-threatening; debilitating; organ failure present								
	Grade IVb	Concomitant multiorgan complications								
	Grade V	Death								
		**Pneumothorax**<br>Collapsed lung; caused by accumulation of air in the pleural cavity								
	Grade I	No intervention required								
	Grade II	Requiring medical therapy only or prolonged (>5 days) pleural drainage		**Pneumothorax Requiring Chest Tube Insertion**<br>Indicate whether the patient experienced a postoperative pneumothorax requiring chest tube reinsertion<br>(Pulmonary)				**Pneumothorax** requiring intervention<br>(Pulmonary)		
	Grade IIIa	Endoscopic, radiological or bedside pleural interventions performed								
	Grade IIIb	Surgical intervention performed under general anaesthesia								
	Grade IVa	Life-threatening; debilitating; organ failure present								
	Grade IVb	Concomitant multiorgan complications								
	Grade V	Death								
		**Prolonged Alveolar Air Leak**<br>Air leak originating from a tear or discontinuity of the visceral pleura lasting > 5 days (i.e. air leak present on POD 6 - surgery is day 0; or air leak is present on discharge from hospital with planned follow-up > POD 6)	**Air Leak > 5 days**<br>Patient experienced a postoperative air leak for > 5 days	**Air Leak > 5 days**<br>Patient experienced a postoperative air leak for > 5 days						
	Grade I	No intervention required								
	Grade II	Requiring medical therapy only or prolonged (>5 days) pleural drainage								
	Grade IIIa	Endoscopic, radiological or bedside pleural interventions performed								
	Grade IIIb	Surgical intervention performed under general anaesthesia								
	Grade IVa	Life-threatening; debilitating; organ failure present								
	Grade IVb	Concomitant multiorgan complications								
	Grade V	Death								
		**Prolonged Pleural Drainage**<br>Tube drainage >5 days						**Chest Tube Drainage** for >10 days post-op<br>(Pulmonary)		
	Grade I	No intervention required								
	Grade II	Requiring medical therapy only or prolonged (>5 days) pleural drainage								
	Grade IIIa	Endoscopic, radiological or bedside pleural interventions performed								
	Grade IIIb	Surgical intervention performed under general anaesthesia								
	Grade IVa	Life-threatening; debilitating; organ failure present								
	Grade IVb	Concomitant multiorgan complications								
	Grade V	Death								
		**Subcutaneous Emphysema**<br>Gas or air present in the subcutaneous layer of the skin or deeper								
	Grade I	No intervention required								
	Grade II	Requiring medical therapy only								
	Grade IIIa	Endoscopic, radiological or bedside pleural interventions performed								
	Grade IIIb	Surgical intervention performed under general anaesthesia								
	Grade IVa	Life-threatening; debilitating; organ failure present								
	Grade IVb	Concomitant multiorgan complications								
	Grade V	Death								
		**Other Pleural Event**								
	Grade I	Transient or equivocal, no therapy added								
	Grade II	Medical therapy added								
	Grade IIIa	Surgical, radiological, endoscopic intervention, or multi-therapy intervention (does not require general anaesthesia)		**Unanticipated Post-Operative Invasive Procedure**<br>Indicate if the patient had an unplanned invasive procedure after surgery. Examples includes return to the operating room for a redo surgical procedure, a percutaneous procedure performed at bedside or in the radiology suite, a tracheostomy, and wound opening at bedside<br>Exclusions: postoperative toilet bronchoscopy, central venous access, arterial line placement, foley catheter placement	**Other events requiring OR with general anesthesia**<br>Indicate whether the patient experienced any other surgical events in the post-operative period requiring a procedure with general anesthesia<br>(Miscellaneous)	**Unexpected Return to the OR**<br>Indicate whether the patient was unexpectedly returned to the OR during this hospital visit				**Unplanned Reoperation**<br>[REOPERATION1]<br>Patient had an unplanned return to the operating room for a surgical procedure related to either the index or concurrent procedure performed; This return must be within the 30 day postoperative period
	Grade IIIb	Surgical intervention performed under general anaesthesia								
	Grade IVa	Leads to single organ failure								
	Grade IVb	Leads to multiple organ failure						**Multisystem Organ Failure**<br>(Other)		
	Grade V	Death								
Pulmonary		**Atelectasis**<br>Part of the lung becomes airless and contracts								
	Grade I	Requiring no intervention other than additional chest physiotherapy								
	Grade II	Requiring endo-tracheal suctioning								
	Grade IIIa	Endoscopic or radiological intervention performed, or non-invasive ventilation (BiPAP) for < 24 hrs	**Bronchoscopy for Atelectasis**<br>Postoperative atelectasis documented clinically or radiographically that needed bronchoscopy	**Atelectasis Requiring Bronchoscopy**<br>Indicate whether the patient experienced atelectasis requiring a bronchoscopy in the postoperative period				**Atelectasis** mucous plugging requiring bronchoscopy<br>(Pulmonary)		
	Grade IIIb	Operative intervention required under general anaesthesia								
	Grade IVa	Respiratory compromise requiring intubation and positive pressure ventilation								
	Grade IVb	Concomitant multiorgan complications								
	Grade V	Death								
		**Adult Respiratory Distress Syndrome (ARDS)**<br>Worsening gas exchange (eg, O2 desaturations [eg, PaO2/FiO2 240], increased oxygen requirements, or increased ventilator demand)<br>Adult respiratory distress syndrome defined according to the American-European consensus conference; all of the following criteria should be met:<br>1. Acute onset<br>2. Arterial hypoxemia with PaO2/FiO2 ratio <200 (regardless of PEEP level)<br>3. Bilateral infiltrates at chest radiograph or CT scan<br>4. No clinical evidence of left atrial hypertension or pulmonary artery occlusive pressure <18 mm Hg<br>5. Compatible risk factors	ARDS	ARDS	**Respiratory Failure**<br>Indicate whether the patient experienced respiratory failure in the postoperative period requiring mechanical ventilation and/or re-intubation			**Acute Respiratory Distress Syndrome (ARDS)**<br>(Pulmonary)<br>3 mutually exclusive categories of ARDS based on degree of hypoxemia:<br>Mild (200 mmHg < PaO2/FiO2 </= 300 mmHg)<br>Moderate (100 mm Hg<PaO2/FiO2 </=200 mmHg)<br>Severe (PaO2/ FiO2 </=100 mmHg)<br>4 ancillary variables for severe ARDS:<br>Radiographic severity<br>Respiratory system compliance (</= 40 mL/cm H2O)<br>Positive end-expiratory pressure (=/>10 cm H2O)<br>Corrected expired volume per minute (=/>10 L/min)		
	Grade I	This complication will not occur at this grade								
	Grade II	Requiring O2 and other medical therapy								
	Grade IIIa	Endoscopic or radiological intervention performed, or non-invasive ventilation (BiPAP) for < 24 hrs								
	Grade IIIb	Operative intervention required under general anaesthesia								
	Grade IVa	Symptomatic and requiring drugs and non-invasive ventilation (e.g. BiPAP) for > 24 hrs; or requiring intubation and ventilation								
	Grade IVb	Concomitant multiorgan complications								
	Grade V	Death								
		**Initial Ventilator Support > 48 hours**<br>Patient was ventilated > 48 hours in the postoperative period; ventilator support ends with removal of the endotracheal tube or, if the patient has a tracheostomy tube, until no longer ventilator dependent	Initial Ventilator Support > 48 hours	Initial Ventilator Support > 48 hours						**Occurrences Ventilator > 48Hours**<br>[FAILWEAN]<br>Total duration of ventilator-assisted respirations during postoperative hospitalization was greater than 48 hours. This can occur at any time during the 30-day period postoperatively. This time assessment is CUMULATIVE, not necessarily consecutive. Ventilator-assisted respirations can be via endotracheal tube, nasotracheal tube, or tracheostomy tube.
	Grade I	This complication will not occur at this grade								
	Grade II	This complication will not occur at this grade								
	Grade IIIa	This complication will not occur at this grade								
	Grade IIIb	This complication will not occur at this grade								
	Grade IVa	Symptomatic and requiring drugs and non-invasive ventilation (e.g. BiPAP) for > 24 hrs; or requiring intubation and ventilation								
	Grade IVb	Concomitant multiorgan complications								
	Grade V	Death								
		**Pneumonia**<br>Defined according to the last CDC criteria: two or more serial chest radiographs with at least one of the following:<br>1. New or progressive and persistent infiltrate<br>2. Consolidation<br>3. Cavitation<br>and at least one of the following:<br>1. Fever (>38 C or >100.4 F) with no other recognized cause<br>2. Leukopenia (<4000 WBC/mm3) or leukocytosis (≥12,000 WBC/mm3)<br>3. For adults >70 years old, altered mental status with no other recognized cause<br>and at least two of the following:<br>1. New onset of purulent sputum, or change in character of sputum, or increased respiratory secretions, or increased suctioning requirements<br>2. New onset or worsening cough, or dyspnea, or tachypnea<br>3. Rales or bronchial breath sounds	Pneumonia	**Pneumonia**<br>Indicate if the patient experienced pneumonia in the postoperative period. Pneumonia is defined as meeting three of five characteristics: fever, leucocytosis, CXR with infiltrate, positive culture from sputum, or treatment with antibiotics<br>(Pulmonary)				**Pneumonia**<br>The presence of a new or progressive radiographic infiltrate plus at least two of three following clinical features.<br>1. Fever greater than 38°C<br>2. Leukocytosis or leukopenia<br>3. Purulent secretions<br>(Pulmonary)		**Occurrences Pneumonia**<br>[OUPNEUMO]<br>Signs/Symptoms/Laboratory:<br>FOR ANY PATIENT, at least one of the following:<br>Fever (>38 C or >100.4 F) with no other recognized cause<br>Leukopenia (<4000 WBC/mm3) or leukocytosis (≥12,000 WBC/mm3)<br>For adults ≥ 70 years old, altered mental status with no other recognized cause<br>AND<br>At least one of the following:<br>5% Bronchoalveolar lavage (BAL) -obtained cells contain intracellular bacteria on direct microscopic exam (e.g., Gram stain)<br>Positive growth in blood culture not related to another source of infection<br>Positive growth in culture of pleural fluid<br>Positive quantitative culture from minimally contaminated lower respiratory tract (LRT) specimen (e.g. BAL or protected specimen brushing)<br>OR<br>At least two of the following:<br>New onset of purulent sputum, or change in character of sputum, or increased respiratory secretions, or increased suctioning requirements<br>New onset or worsening cough, or dyspnea, or tachypnea<br>Rales or rhonchi<br>Worsening gas exchange (e.g. O2 desaturations (e.g., PaO2/FiO2 ≤ 240), increased oxygen requirements, or increased ventilator demand)<br>Radiology:<br>One definitive chest radiological exam (x-ray or CT)* with at least one of the following:<br>New or progressive and persistent infiltrate<br>Consolidation or opacity<br>Cavitation
	Grade I	This complication will not occur at this grade								
	Grade II	Requiring antibiotic therapy								
	Grade IIIa	Endoscopic or radiological intervention performed, or non-invasive ventilation (BiPAP) for < 24 hrs								
	Grade IIIb	Operative intervention required under general anaesthesia								
	Grade IVa	Life-threatening consequences (e.g., septic shock, respiratory failure, or hypotension)								
	Grade IVb	Concomitant multiorgan complications								
	Grade V	Death								
		**Other Pulmonary Event**		**Other Pulmonary Event**<br>Indicate whether another pulmonary event occurred in the postoperative period						
	Grade I	Transient or equivocal, no therapy added								
	Grade II	Medical therapy added								
	Grade IIIa	Surgical, radiological, endoscopic intervention, or multi-therapy intervention (does not require general anaesthesia)			**Unanticipated Post-Operative Invasive Procedure**<br>Indicate if the patient had an unplanned invasive procedure after surgery. Examples includes return to the operating room for a redo surgical procedure, a percutaneous procedure performed at bedside or in the radiology suite, a tracheostomy, and wound opening at bedside<br>Exclusions: postoperative toilet bronchoscopy, central venous access, arterial line placement, foley catheter placement	**Other events requiring OR with general anesthesia**<br>Indicate whether the patient experienced any other surgical events in the post-operative period requiring a procedure with general anesthesia<br>(Miscellaneous)	**Unexpected Return to the OR**<br>Indicate whether the patient was unexpectedly returned to the OR during this hospital visit			**Unplanned Reoperation**<br>[REOPERATION1]<br>Patient had an unplanned return to the operating room for a surgical procedure related to either the index or concurrent procedure performed; This return must be within the 30 day postoperative period
	Grade IIIb	Surgical intervention performed under general anaesthesia								
	Grade IVa	Leads to single organ failure								
	Grade IVb	Leads to multiple organ failure						**Multisystem Organ Failure**<br>(Other)		
	Grade V	Death								
Renal		**Cystitis**<br>Inflammation of the urinary bladder due to infection								
	Grade I	Asymptomatic, intervention not indicated								
	Grade II	Requiring medical therapy only (e.g. bladder irrigation)								
	Grade IIIa	Requiring transfusion; IV pain medications								
	Grade IIIb	Catastrophic bleeding; major non-elective intervention indicated								
	Grade IVa	Life-threatening; debilitating; organ failure present								
	Grade IVb	Concomitant multiorgan complications								
	Grade V	Death								
		**Renal Failure**<br>Defined as the onset of new renal failure in the postoperative period according to one of the following criteria:<br>1. Increase of serum creatinine to >2.0 mg/dL<br>2. Two times the preoperative creatinine level<br>3. A new requirement for dialysis postoperatively	Renal Failure	**Renal Failure - RIFLE Criteria**<br>Indicate whether the patient had acute renal failure or worsening renal function resulting in ONE OR BOTH of the following:<br>1. Increase in serum creatinine level 3.0 x greater than baseline, or serum creatinine level >4 mg/dL. Acute rise must be at least 0.5 mg/dL.<br>2. A new requirement for dialysis postoperatively.<br>(Miscellaneous)				**Acute Renal Insufficiency**<br>Doubling of baseline creatinine<br>(Urologic)		**Occurrences Progressive Renal Insufficiency**<br>[RENAINSF]<br>The reduced capacity of the kidney to perform its function as evidenced by a rise in creatinine of >2 mg/dl from preoperative value, but with no requirement for dialysis within 30 days of the operation
	Grade I	No intervention required								
	Grade II	Requiring medical therapy only (e.g. diuretics)								
	Grade IIIa	Requiring dialysis							**Acute renal failure** requiring dialysis<br>(Urologic)	**Occurrences Acute Renal Fail**<br>[OPRENAFL]<br>A patient who did not require dialysis preoperatively, worsening of renal dysfunction postoperatively requiring hemodialysis, peritoneal dialysis, hemofiltration, hemodiafiltration, or ultrafiltration.<br>If the patient refuses a recommendation for dialysis, you would answer 'Yes' to this variable because the patient required dialysis.<br>Hemodialysis, peritoneal dialysis, hemofiltration, hemodiafiltration, or ultrafiltration all qualify.<br>Placement of a dialysis catheter is indicative of the need for dialysis, if used within 48 hours of placement, would not be assigned
	Grade IIIb	Surgical intervention performed under general anaesthesia								
	Grade IVa	Life-threatening; debilitating; organ failure present								
	Grade IVb	Concomitant multiorgan complications								
	Grade V	Death								
		**Urinary Retention**<br>Lack of ability to urinate								
	Grade I	No intervention required								
	Grade II	Requires short term re- catheterization (<72 hrs)		**Urinary Retention**<br>Indicate whether the patient experienced urinary retention requiring catheterization<br>(Urologic)<br>**Discharged with a Foley Catheter**<br>Indicate whether the patient was discharged with a Foley Catheter in place<br>(Urologic)				**Urinary retention** requiring reinsertion of urinary catheter, delaying discharge or discharge with urinary catheter<br>(Urologic)		
	Grade IIIa	Requires long term re- catheterization (>72 hrs) or D/C home with catheter								
	Grade IIIb	Surgical intervention performed under general anaesthesia								
	Grade IVa	Life-threatening; debilitating; organ failure present								
	Grade IVb	Concomitant multiorgan complications								
	Grade V	Death								
		**Urinary Tract Infection (UTI)**<br>Bacterial infection that affects any part of the urinary tract								**Occurrences Urinary Tract Infection**<br>[URNINFEC]<br>An infection in the urinary tract (kidneys, ureters, bladder, and urethra). Must be noted within 30 days after the principal operative procedure AND patient must meet ONE of the following:<br>A: ONE of the following six criteria:<br>Fever (>38oC or 100.4o F)<br>Urgency<br>Frequency<br>Dysuria<br>Suprapubic tenderness<br>Costovertebral angle pain or tenderness<br>AND<br>A urine culture of > 100,000 colonies/ml urine with no more than two species of organisms. Signs and symptoms should be reported within 72 hours prior to a urine culture being sent or 24 hours after the culture was sent<br>[illegible]
	Grade I	No intervention required other than removal of foley								
	Grade II	Requiring medical therapy only (e.g. antibiotics)								

	Grade IIIa	Endoscopic, radiological or bedside interventions performed		**Urinary Tract Infection**<br>Indicate if the patient experienced a urinary tract infection (with positive urine cultures postoperatively) requiring treatment<br>(Urologic)				**Urinary Tract Infection***<br>(Urologic)		OR<br>B: TWO of the following six criteria:<br>Fever (>38o C or 100.4o F)<br>Urgency<br>Frequency<br>Dysuria<br>Suprapubic tenderness<br>Costovertebral angle pain or tenderness<br>AND<br>At least one of the following:<br>Dipstick test positive for leukocyte esterase and/or nitrate<br>Pyuria (>10 WBCs/mm3 or > 3 WBC/hpf of unspun urine)<br>Organisms seen on Gram stain of unspun urine<br>Two urine cultures with repeated isolation of the same uropathogen with >100,000 colonies/ml urine in non-voided specimen. Signs and symptoms should be reported within 72 hours prior to a urine culture being sent or 24 hours after the culture was sent. Urine culture with < 100,000 colonies/ml urine of single uropathogen in patient being treated with appropriate antimicrobial therapy. Signs and symptoms should be reported within 72 hours prior to a urine culture being sent or 24 hours after the culture was sent<br>Physician's diagnosis<br>Physician institutes appropriate antimicrobial therapy		
	Grade IIIb	Surgical intervention performed under general anaesthesia										
	Grade IVa	Life-threatening; debilitating; organ failure present										
	Grade IVb	Concomitant multiorgan complications										
	Grade V	Death										
		**Other Renal Event**										
	Grade I	Transient or equivocal, no therapy added										
	Grade II	Medical therapy added										
	Grade IIIa	Surgical, radiological, endoscopic intervention, or multi-therapy intervention (does not require general anaesthesia)		**Unanticipated Post-Operative Invasive Procedure**<br>Indicate if the patient had an unplanned invasive procedure after surgery. Examples includes return to the operating room for a redo surgical procedure, a percutaneous procedure performed at bedside or in the radiology suite, a tracheostomy, and wound opening at bedside Exclusions: postoperative toilet bronchoscopy, central venous access, arterial line placement, foley catheter placement								
	Grade IIIb	Surgical intervention performed under general anaesthesia			**Other events requiring OR with general anesthesia**<br>Indicate whether the patient experienced any other surgical events in the post-operative period requiring a procedure with general anesthesia<br>(Miscellaneous)	**Unexpected Return to the OR**<br>Indicate whether the patient was unexpectedly returned to the OR during this hospital visit				**Unplanned Reoperation**<br>[REOPERATION1]<br>Patient had an unplanned return to the operating room for a surgical procedure related to either the index or concurrent procedure performed; This return must be within the 30 day postoperative period		
	Grade IVa	Leads to single organ failure										
	Grade IVb	Leads to multiple organ failure						**Multisystem Organ Failure**<br>(Other)				
	Grade V	Death										
Wound		**Dehiscence**<br>Previously closed wound reopening						**Thoracic Wound Dehiscence***<br>(Wound/Diaphragm)	**Acute Abdominal Wall Dehiscence/Hernia***<br>(Wound/Diaphragm)	**Occurrences Wound Disrupt**<br>[DEHIS]<br>The spontaneous reopening of a previously surgically closed wound that occurs within 30 days after the principal operative procedure AND one of the following criteria:<br>Abdominal site: refers primarily to loss of the integrity of fascial closure (or whatever closure was performed in the absence of fascial closure)<br>OR<br>Other Surgical Sites: there must be a total breakdown of the surgical closure compromising the integrity of the procedure		
	Grade I	This complication will not occur at this grade										
	Grade II	This complication will not occur at this grade										
	Grade IIIa	Requiring intervention										
	Grade IIIb	Surgical intervention performed under general anaesthesia										
	Grade IVa	Concomitant multiorgan complications										
	Grade IVb	Life-threatening consequences; major urgent intervention indicated										
	Grade V	Death										
		**Hematoma**<br>Collection of blood outside the blood vessels, generally the result of hemorrhage										
	Grade I	No intervention required										
	Grade II	Transfusion, evacuation or aspiration. Opening of wound at bedside										
	Grade IIIa	Interventional radiology indicated										
	Grade IIIb	Surgical intervention performed under general anaesthesia										
	Grade IVa	Concomitant multiorgan complications										
	Grade IVb	Life-threatening consequences; major urgent intervention indicated										
	Grade V	Death										
		**Infection**<br>Patient experienced a wound infection in the postoperative period as evidenced by meeting two of the following criteria:<br>1. Wound opened with excision of tissue (I&D)<br>2. Positive culture<br>3. Treatment with antibiotics	Wound Infection	**Surgical Site Infection (Superficial, Deep, Organ space)**<br>Indicate the extent of surgical site infection if one was present within 30 days of surgery<br>(Infection)								
	Grade I	This complication will not occur at this grade										
	Grade II	Requiring medical therapy (e.g. antibiotics) or opening wound						**Wound infection** requiring opening wound or antibiotics<br>(Infection)		**Superficial Incisional Surgical Site Infection** (SSI)<br>[SUPINFEC]<br>Superficial incisional SSI is an infection that occurs within 30 days after the operation and the infection involves only skin or subcutaneous tissue of the incision and at least one of the following:<br>Purulent drainage, with or without laboratory confirmation, from the superficial incision<br>Organisms isolated from an aseptically obtained culture of fluid or tissue from the superficial incision<br>At least one of the following signs or symptoms of infection: pain or tenderness, localized swelling, redness, or heat AND superficial incision is deliberately opened by the surgeon, unless incision is culture-negative or the physician documented NO infection and does not further treat the patient<br>Diagnosis of superficial incisional SSI by the surgeon or attending physician	**Occurrences Deep Incisional SSI**<br>[WNDINFD]<br>Deep incision SSI is an infection that occurs within 30 days after the operation and the infection appears to be related to the operation and infection involved deep soft tissues (e.g., fascial and muscle layers) of the incision and at least one of the following:<br>Purulent drainage from the deep incision but not from the organ/space component of the surgical site<br>A deep incision spontaneously dehisces or is deliberately opened by a surgeon when the patient has at least one of the following signs or symptoms: Fever (> 38 C), localized pain, or tenderness, unless site is culture-negative<br>An abscess or other evidence of infection involving the deep incision is found on direct examination, during reoperation, or by histopathologic or radiologic examination<br>Diagnosis of a deep incision SSI by a surgeon or attending physician	**Occurrences Organ Space SSI**<br>[ORGSPCSSI]<br>Organ/Space SSI is an infection that occurs within 30 days after the operation and the infection appears to be related to the operation and the infection involves any part of the anatomy (e.g., organs or spaces), other than the incision, which was opened or manipulated during an operation and at least one of the following:<br>Purulent drainage from a drain that is placed through a stab wound into the organ/space<br>Organisms isolated from an aseptically obtained culture of fluid or tissue in the organ/space<br>An abscess or other evidence of infection involving the organ/space that is found on direct examination, during reoperation, or by histopathologic or radiologic examination<br>Diagnosis of an organ/space SSI by a surgeon or attending physician
	Grade IIIa	Wound opened/explored at bedside										
	Grade IIIb	Surgical intervention performed under general anaesthesia										
	Grade IVa	Concomitant multiorgan complications										
	Grade IVb	Life-threatening; debilitating; organ failure present										
	Grade V	Death										
		**Seroma**<br>Pocket of clear serous fluid that develops in the body after surgery										
	Grade I	No intervention required										
	Grade II	Opening of wound at bedside										
	Grade IIIa	Transfusion, interventional radiology indicated										
	Grade IIIb	Surgical intervention performed under general anaesthesia										
	Grade IVa	This complication will not occur at this grade										
	Grade IVb	This complication will not occur at this grade										
	Grade V	This complication will not occur at this grade										
		**Other Wound Event**										
	Grade I	Transient or equivocal, no therapy added										
	Grade II	Medical therapy added										
	Grade IIIa	Surgical, radiological, endoscopic intervention, or multi-therapy intervention (does not require general anaesthesia)		**Unanticipated Post-Operative Invasive Procedure**<br>Indicate if the patient had an unplanned invasive procedure after surgery. Examples includes return to the operating room for a redo surgical procedure, a percutaneous procedure performed at bedside or in the radiology suite, a tracheostomy, and wound opening at bedside Exclusions: postoperative toilet bronchoscopy, central venous access, arterial line placement, foley catheter placement								
	Grade IIIb	Surgical intervention performed under general anaesthesia			**Other events requiring OR with general anesthesia**<br>Indicate whether the patient experienced any other surgical events in the post-operative period requiring a procedure with general anesthesia<br>(Miscellaneous)	**Unexpected Return to the OR**<br>Indicate whether the patient was unexpectedly returned to the OR during this hospital visit				**Unplanned Reoperation**<br>[REOPERATION1]<br>Patient had an unplanned return to the operating room for a surgical procedure related to either the index or concurrent procedure performed; This return must be within the 30 day postoperative period		
	Grade IVa	Leads to single organ failure										
	Grade IVb	Leads to multiple organ failure						**Multisystem Organ Failure**<br>(Other)				
	Grade V	Death										
Complication Modifier		**Resulted in Reintubation**<br>Any complication, Grade IVa or above that resulted in reintubation	**Reintubation**<br>Patient was reintubated during the initial hospital stay after the initial extubation; this may include patients who have been extubated in the operating room and require intubation in the postoperative period	**Reintubation**<br>Patient was reintubated during the initial hospital stay after the initial extubation; this may include patients who have been extubated in the operating room and require intubation in the postoperative period				Respiratory failure requiring **reintubation**<br>(Pulmonary)		**Occurences Unplanned Intubation**<br>[REINTUB]<br>Patient required placement of an endotracheal tube or other similar breathing tube (Laryngeal Mask Airway (LMA), nasotracheal tube, etc) and ventilator support intraoperatively or within 30 days following surgery which was not intended or planned		
	Grade I	This complication will not occur at this grade										
	Grade II	This complication will not occur at this grade										
	Grade IIIa	This complication will not occur at this grade										
	Grade IIIb	This complication will not occur at this grade										
	Grade IVa	Concomitant multiorgan complications										
	Grade IVb	Life-threatening consequences; major urgent intervention indicated										
	Grade V	Death										
		**Resulted in Tracheostomy**<br>Any complication, Grade IIIb or above that resulted in tracheostomy	**Tracheostomy**<br>Patient required a tracheostomy in the postoperative period whether performed in the ICU or the OR; prophylactic minitracheostomy on the day of operation should not be considered a complication<br>(Pulmonary)	**Tracheostomy**<br>Patient required a tracheostomy in the postoperative period whether performed in the ICU or the OR								
	Grade I	This complication will not occur at this grade										
	Grade II	This complication will not occur at this grade										
	Grade IIIa	This complication will not occur at this grade										
	Grade IIIb	Surgical intervention performed under general anaesthesia										
	Grade IVa	Concomitant multiorgan complications										
	Grade IVb	Life-threatening consequences; major urgent intervention indicated										
	Grade V	Death										
		**Resulted in Unplanned Transfer to ICU**<br>Any complication, Grade IVa or above that resulted in unplanned transfer to ICU	**Unexpected Admission to ICU**<br>An unplanned transfer of the patient to the ICU owing to deterioration in the condition of the patient requiring active life support treatment	**Unexpected Admission to ICU**<br>Indicate whether there was an unplanned transfer of the patient to the ICU due to deterioration in the condition of the patient								
	Grade I	This complication will not occur at this grade										
	Grade II	This complication will not occur at this grade										
	Grade IIIa	This complication will not occur at this grade										
	Grade IIIb	This complication will not occur at this grade										
	Grade IVa	Concomitant multiorgan complications										
	Grade IVb	Life-threatening consequences; major urgent intervention indicated										
	Grade V	Death										
		**Resulted in Readmission**		**Readmission Related To Operative Procedure**<br>Indicate whether the readmission was related to this operation<br>(Discharge)<br>**Readmission within 30 days of Discharge**<br>Indicate whether patient was readmitted to any hospital within 30 days of discharge<br>(Discharge)						**Readmission**<br>[READMISSION]<br>Any readmission (to the same or another hospital), for any reason, within 30 days of the principal surgical procedure		
		**Prolonged Length of Stay**										
		**Resulted in Return to Emerg or Clinic**										
		**Resulted in Unplanned Transfer to Step-Down Unit**										
		**Complication Not Related to Procedure**										
Intraoperative Complications		Arrest (Cardiac)										
		CVA										
		Clinically Significant Bleeding										
		Clinically Significant Bleeding - Requiring Transfusion										
		Death										
		Injury to Airway		**Tracheobronchial Injury**<br>Indicate whether a tracheobronchial injury occurred				**Tracheobronchial Injury**				
		Injury to Esophagus										
		Loss of Normal Thermia (< 35 C)										
		Myocardial Ischemia										
		Staple Misfire										
		Unstable Arrythmia										
