# Supplementary material for: Harmonization of adverse events monitoring following thoracic surgery: Pursuit of a common language and methodology
Source: JTCVS Open. 2021 Apr 2;6:250–6. doi: 10.1016/j.xjon.2021.03.021 (PMC9390191; doi:10.1016/j.xjon.2021.03.021)
Supplement: Appendix 2 [file mmc2.pdf]

Table 2: AEs not harmonized with CATS database

Classification System	STS 4.1[3]	ECCG[4]	NSQIP[5,6]
Type of Complication (CATS classifications)	Complication	Complication	Complication
Cardiovascular		**Cardiac Arrest Requiring CPR**<br>(Cardiac)	**Occurrences Cardiac Arrest Requiring CPR**<br>[CDARREST]<br>The absence of cardiac rhythm or presence of chaotic cardiac rhythm, intraoperatively or within 30 days following surgery, which results in a cardiac arrest requiring the initiation of CPR, which includes chest compressions
Gastrointestional		**Reoperation for Abdominal Bleeding**<br>(Other)	
		**Acute Diaphragmatic Hernia**<br>(Wound/Diaphragm)	
	**Clostridium Difficile Infection**	**Clostridium Difficile Infection**	**Occurrences Clostridium Difficile Colitis**<br>[OTHCDIFF]<br>Postoperative Clostridium Difficile (C. diff) Colitis
	**Dilation of The Esophagus**<br>Indicate whether the patient required dilation of the esophagus within the postoperative period		
		**Feeding J-tube Complication**	
		**Liver Dysfunction**	
		**Pancreatitis**	
		**Pyloromyotomy/Pyloroplasty Complication**	
		**Small Bowel Obstruction**	
Neurological		**Delirium Tremens**<br>(Neurologic/Psychiatric)	
Pleural	**Discharged With Chest Tube**<br>Indicate whether the patient was discharged with a chest tube for persistent air leak or to drain a postoperative effusion<br>(Discharge)		
Pulmonary	**Discharged With Home O2**<br>New; not using O2 pre-op<br>(Discharge)		
		**Acute Aspiration**	
System			**Other postoperative surgical occurrence**<br>[PODIAG_OTHER]<br>Other postoperative surgical occurrences which are significant and that are not covered by other postoperative outcome criteria
Wound	**Other Infection Requiring IV Antibiotics**<br>Indicate whether the patient experienced any other infection requiring IV antibiotics<br>(Infection)	**Other infections requiring antibiotics** (Infection)	
	**Sepsis**<br>Indicate whether the patient experienced sepsis (septicemia) requiring positive blood cultures in the postoperative period<br>(Infection)	**Generalized sepsis** – CDC definition<br>(Infection)	**Occurences sepsis**<br>[OTHSYSEP]<br>Sepsis is the systemic response to infection. Report this variable if the patient has two of the following clinical signs and symptoms of SIRS:<br>Temp >38o C (100.4 o F) or < 36 o C (96.8 o F)<br>HR >90 bpm<br>RR >20 breaths/min or PaCO2 <32 mmHg(<4.3 kPa)<br>WBC >12,000 cell/mm3, <4000 cells/mm3, or >10% immature (band) forms<br>Anion gap acidosis: this is defined by either: [Na + K] – [Cl + HCO3 (or serum CO2)]. If this number is greater than 16, then an anion gap acidosis is present. Na – [Cl + HCO3 (or serum CO2)]. If this number is greater than 12, then an anion gap acidosis is present. *If anion gap lab values are performed at your facilities lab, ascertain which formula is utilized and follow guideline criteria.<br><br>And either A or B below:<br><br>A. One of the following:<br>Positive blood culture<br>Clinical documentation of purulence or positive culture from any site for which there is documentation noting the site as the acute cause of sepsis<br><br>B. The patient must meet SIRS criteria within 48 hours after the Principal Operative Procedure AND One of the following findings during the Principal Operative Procedure:<br>Confirmed infarcted bowel requiring resection<br>Purulence in the operative site<br>Enteric contents in the operative site, or<br>Positive intra-operative cultures
	**Postoperative Packed Red Blood Cells**<br>Indicate whether the patient received packed Red Blood Cells (RBC) postoperatively<br>(Hematology)		**Occurrences Bleeding Transfusions**<br>[OTHBLEED]<br>At least 1 unit of packed or whole red blood cells given from the surgical start time up to and including 72 hours postoperatively
		**Central IV line infection** requiring removal or antibiotics<br>(Infection)	
		**Intrathoracic/Intra-abdominal Abscess**<br>(Infection)	
			**Occurrences Septic Shock**<br>[OTHSESHOCK]<br>Sepsis is considered severe when it is associated with organ and/or circulatory dysfunction. Report this variable if the patient has the clinical signs and symptoms of SIRS or sepsis AND documented organ and/or circulatory dysfunction
