# Supplementary figures and images for: Harmonization of adverse events monitoring following thoracic surgery: Pursuit of a common language and methodology
Source: JTCVS Open. 2021 Apr 2;6:250–6. doi: 10.1016/j.xjon.2021.03.021 (PMC9390191; doi:10.1016/j.xjon.2021.03.021)

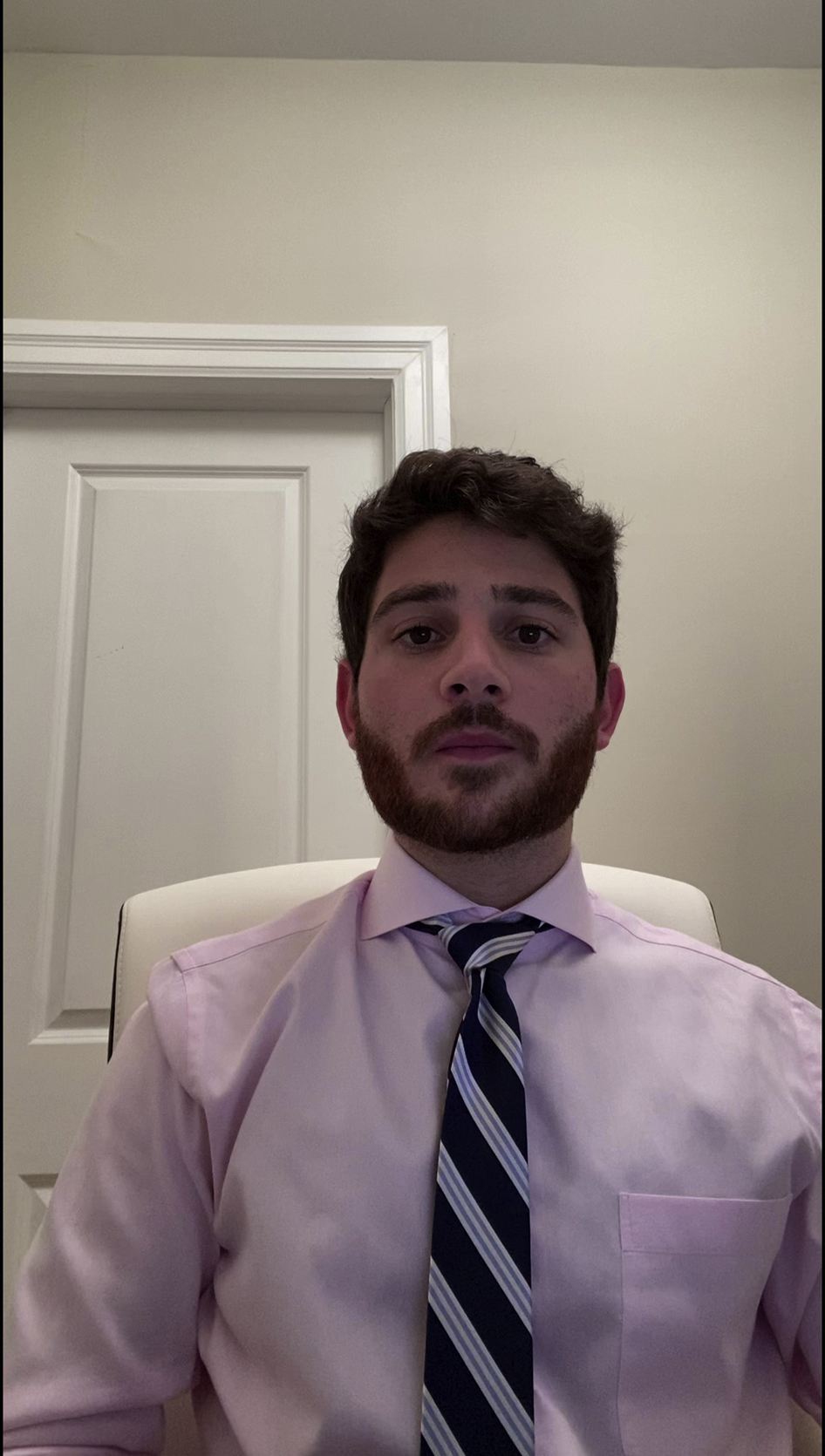

Supplement: Video 1 — Video recording of primary author (Gregory Sigler) describing the purpose, methodology, outcomes and implications of the project. Video available at: https://www.jtcvs.org/article/S2666-2736(21)00078-4/fulltext. [file fx3.jpg]
